# “Unite for safety – clean your hands”: the 5 May 2022 World Health Organization *SAVE LIVES—Clean Your Hands* campaign

**DOI:** 10.1186/s13756-022-01105-y

**Published:** 2022-04-29

**Authors:** Ermira Tartari, Claire Kilpatrick, Benedetta Allegranzi, Didier Pittet

**Affiliations:** 1 Infection Prevention and Control Technical and Clinical Hub, Department of Integrated Health Services, World Health Organization (WHO), Geneva, Switzerland; 2 Faculty of Health Sciences, University of Malta, Msida, Malta; 3 Infection Control Program, Hospitals and Faculty of Medicine, University of Geneva, Gabrielle-Perret-Gentil 4, 1205 Geneva, Switzerland

Hand hygiene improvement is a critical part of effective infection prevention and control (IPC) and therefore constitutes a priority for patient and health worker’s safety. However, hand hygiene compliance in healthcare settings remains sub-optimal globally [1–3]. The World Health Organization (WHO) recommends to implement an effective [4] Multimodal Hand Hygiene Improvement Strategy (MMIS) that includes five elements: i) system change; ii) training and education; iii) monitoring and feedback; iv) reminders in the workplace/communications; v) safety climate/culture change [4].

Systematic reviews have shown an inter-relation between safety culture, IPC processes and healthcare-associated infection (HAI) reduction [5, 6]. Improving the organizational safety climate has been associated with enhanced hand hygiene compliance [7–10] and improved patient outcomes, including HAI reduction [11, 12], in particular *vancomycin-resistant enterococci* and *Staphylococcus aureus* [10, 13] and central line-associated bloodstream [14, 15] infections.

Employing the Hand Hygiene Self-Assessment Framework (HHSAF) [16] to assess the implementation of the WHO MMIS in healthcare facilities worldwide, the *Institutional Safety Climate* element repetitively scored the lowest [17, 18], suggesting that progress in improving safety climate has been slower across and within regions when compared with the four other elements of the MMIS. Therefore, it seems critical to direct attention to safety climate/culture change to ensure further and sustainable hand hygiene improvement. Safety climate, safety culture and organizational culture are often used interchangeably whereas their concepts are distinct. Organizational culture refers to the deeply embedded norms, values, beliefs, and assumptions shared by members within an organization [19]. Safety culture considers leadership and health workers attitudes and values related to the perception of risk and safety. Safety climate is a subset of overall organizational climate that refers to employees’ perceptions about the extent to which the organization values safety (for patients, health workers and the environment) [12, 19]. The *Institutional Safety Climate* as part of the hand hygiene MMIS refers to the environment and perceptions of patient safety issues in a healthcare facility in which hand hygiene improvement is given high priority and valued at all levels of the organization [20]. This includes the perception and belief that resources are provided and available to ensure hand hygiene, particularly at the point of care. In summary, when a health facility's "quality and safety climate or culture" values hand hygiene and IPC, this results in both patients and health workers feeling protected and cared for. To prioritize clean hands at the point of care at the right times using the right agent and technique, people at all levels, including those using healthcare facilities, should focus on the importance of hand hygiene to save lives and act as key players in achieving and promoting the appropriate behaviors and attitudes towards it.

In light of the importance of this element and given the limited progress made in the last 20 years, the World Hand Hygiene Day, 5 May 2022, promotes institutional safety climate/culture change as a priority for hand hygiene improvement by adopting the slogan “Unite for safety – clean your hands” (Fig. 1). To achieve unity for safety, WHO calls all key stakeholders to participate actively (Table 1).

**Fig. 1:**
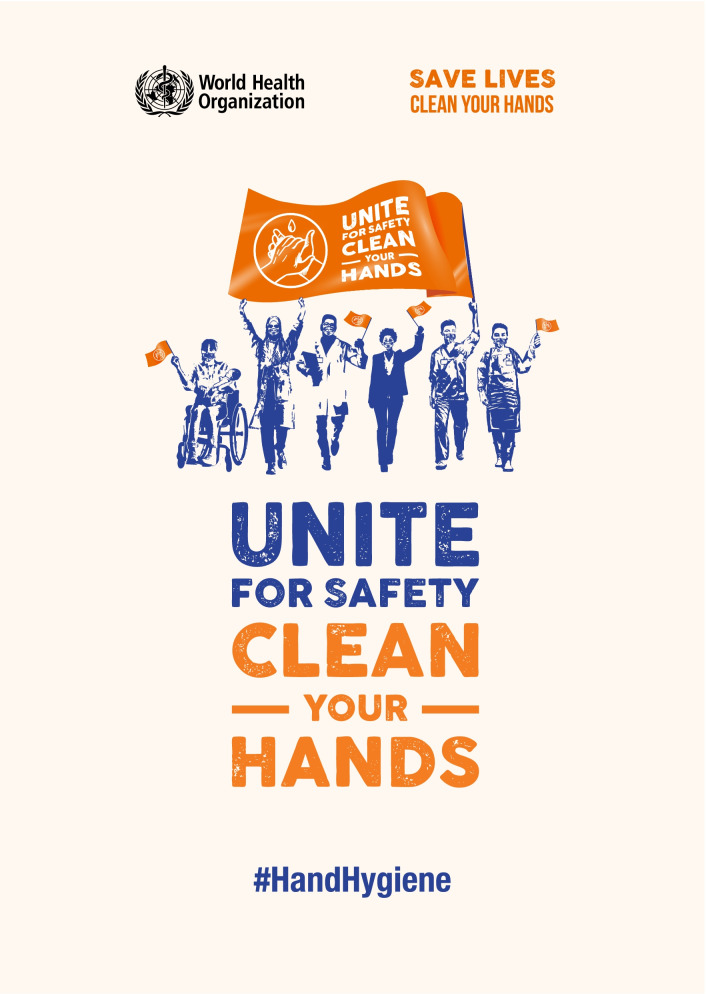
5 May 2022: “Unite for safety—clean your hands”. 5 May 2022 WHO *SAVE LIVES: Clean Your Hands* campaign slogan and main promotional image (2022 hashtag: #HandHygiene)

**Table 1:** 5 May 2022 *WHO SAVE LIVES: Clean Your Hands* campaign calls to action

Campaign participants	Call to action
Health care workers	“Thank you for leading by example and encouraging others to clean their hands”
IPC* practitioners	“Thank you for engaging health workers to be part of new hand hygiene initiatives”
Quality and safety leads	“Thank you for working with infection prevention colleagues to support hand hygiene improvement”
Facility managers	“Thank you for promoting a quality and safety culture to ensure clean hands”
Policy-makers	“Thank you for prioritizing resources, training and programmes on hand hygiene”
People who use health care	“Thank you for getting involved in local hand hygiene campaigns and activities”

IPC: infection prevention and control

Healthcare facilities can use the HHSAF [16] to track the level of progress with hand hygiene implementation, including safety climate and culture change, evaluating improvement over time. This tool also helps to develop an action plan to ensure long-term sustainability. Factors ultimately required to create and support an environment that raises awareness about patient safety and quality of care while ensuring that hand hygiene best practices are prioritized at all levels include: i) a team dedicated to the promotion and implementation of hand hygiene in the facility; ii) leadership commitment and active participation, ii) promotional activities; iii) champions and role models; iv) engagement of patients and patient organizations; v) institutional targets, accountability and reporting. Additionally, self-efficacy and individual accountability should be supported in the organization as well as nurturing of role models and champions at every level.

We call on the international community to get involved in the World Hand Hygiene Day 2022 (https://www.who.int/campaigns/world-hand-hygiene-day/2022) and work together to accelerate progress across health services. Reaffirm your commitment, unite, talk and work together on hand hygiene for future progress, sustainability and ultimately improved quality and safer care: “Unite for safety—Clean your hands!”.

## Data Availability

All the information is available on the webpage WHO *SAVE LIVES: Clean Your Hands* campaign and World Hand Hygiene Day 2022 (https://www.who.int/campaigns/world-hand-hygiene-day/2022), including an advocacy toolkit offering guidance on the campaign’s objectives, key messages and how to get involved.
